# Physeal Allograft Transfer for Physeal Bars: A Safety and Feasibility Study in a Domestic Swine Model

**DOI:** 10.1002/jor.70133

**Published:** 2026-01-04

**Authors:** J. V. Korpershoek, C. Chen, C. V. Nagelli, K. L. Lydon, M. L. Floren, D. B. F. Saris, A. N. Larson, T. A. Milbrandt

**Affiliations:** ^1^ Department of Orthopedic Surgery Mayo Clinic Rochester MN USA; ^2^ Department of Orthopaedic Surgery University Medical Center Utrecht Utrecht the Netherlands; ^3^ Department of Orthopedics Ditmanson Medical Foundation Chia‐yi Christian Hospital Chia‐yi Taiwan; ^4^ Department of Orthopedic Surgery National Taiwan University Hospital Taipei Taiwan; ^5^ School of Medicine National Taiwan University Taipei Taiwan; ^6^ Innovation Department AlloSource Centennial CO USA

**Keywords:** allograft, growth plate, in vivo, Physis, transfer

## Abstract

Premature physeal closure occurs following trauma, cancer, or infection. Current treatments have poor success rates. With recent pediatric donor tissue availability, physeal allograft transfer (PAT) can now be considered. The purpose of this study was to study the safety and feasibility of PAT in a large animal model. The aim of this study is to gather foundational data to inform future studies into the efficacy of PAT. Physeal defects were created in the distal femur of nine female domestic swine and treated with PAT from two male donor pigs, cementation, or bone autograft. Viability was assessed. After 3 months, physes were visualized using CT and MRI. Integration, tissue composition, donor DNA presence, and microscopic appearance were evaluated. Physeal allografts demonstrated 93% viability after procurement and preservation. All animals reached the 3‐months study endpoint without gross deformations. No physeal bars formed in any group. Cystic changes were seen in experimental and control femurs. All groups showed disorganized tissue architecture without growth plate recapitulation. High allografts viability and structural integrity after procurement support the potential of this treatment. Although no gross deformities were found, transferred physes demonstrate poor integration and incomplete repair. The lack of physeal bar formation in the control group limits the reliability of the animal model for studying physeal allograft transfer. Lastly, this study was designed as a feasibility study and lacks power to compare treatment effects statistically. The efficacy of PAT for preventing growth arrest remains undetermined.

## 1 Introduction

The growth plate, or physis, is a cartilaginous structure located near the ends of long bones that creates bone length and growth until its closure at adolescence. Damage to the physis in pediatric patients can be caused by trauma, tumor growth, or infection [1, 2, 3]. If damage results in osseous consolidation from metaphysis to epiphyses, the resulting physeal bar can tether the growth and cause length discrepancies, or angular and rotational deformities [4]. Treatment of physeal bars has been consistent over the last decades, and is dependent on age. Removal of the opposite limb growth plate (epiphysiodesis) or conservative management are preferred treatments for older children with minimal residual growth [5]. In younger patients with substantial growth potential, treatment of physeal bars consists of excision of the bar under direct visualization and interposing methylmethacrylate or fat to prevent further angulation or deformity, while allowing growth [5]. However, these treatments have a limited success rate [6] and do not restore the growth potential of the lost physis. For decades, physeal transfer was attempted by physicians and researchers to maintain growth potential using revascularized grafts, which resulted in good but inconsistent outcomes. Furthermore, transfer of revascularized grafts is surgically demanding and technically challenging, with long surgery times to obtain optimal anastomosis, and high risks of thrombosis [7]. In addition, physeal autografts cause donor site morbidity and have different growth potentials when applied heterotopically. Physeal vascularized allografts require immuned suppression which caused considerable toxicity and side effects. In addition, physeal vascularized allograft transfer resulted in an early allergic reaction to the allogeneic vasculature [7].

The cartilage that makes up the physis itself consists of a dense tissue and therefore enjoys a relative immune privileged status, as the extracellular matrix cannot be penetrated easily by immune cells [8]. As such, osteochondral allograft (OCA) transplantation is performed routinely for osteochondral defects, and have high survival rates of up to 82% at 10 year [9]. Despite pediatric donor physes readily available from tissue banks, unvascularized physeal allograft transfer has not been pursued for pediatric patients with physeal defects. Given the high success rates of fresh OCAs transplantation and the significant work performed to optimize tissue viability in OCAs [10], successfully adapting these procedures for treatment of physeal defects in a pediatric population would have a high clinical impact and straightforward clinical translation. Therefore, we performed a safety and feasibility study to assess the feasibility and safety of a physeal allograft transfer in a large animal model. In this study, we employed a domestic pig model to ensure adequate axial limb growth during the follow‐up period [11]. Furthermore, the relatively great physeal height in pigs was taken into account to facilitate optimal alignment with the surrounding physeal tissue at the time of surgical transfer. Our hypothesis was that the cartilage cells within the physis would be viable and that a transferred physeal allograft would restore growth.

## 2 Materials and Methods

### 2.1 Ethical Considerations

All procedures involving animals were reviewed and approved by the Institutional Animal Care and Use Committee (IACUC) at Mayo Clinic, Rochester, MN, USA. The study protocol was established before the study, but not published publicly. Immature domestic swine were selected as the preclinical animal model due to the size of their physis, physiological similarities to human physis, and substantial expected growth in feasible time for in vivo studies. All surgical procedures were performed under appropriate anesthesia, and analgesia was administered to minimize pain and distress. Postoperative monitoring protocols were strictly followed to assess animal welfare, and humane endpoints were established before starting the study (supplemental information). The study adhered to the ARRIVE guidelines and authors have supplied the ARRIVE Checklist.

### 2.2 Study Design and Groups

**Figure 1:**
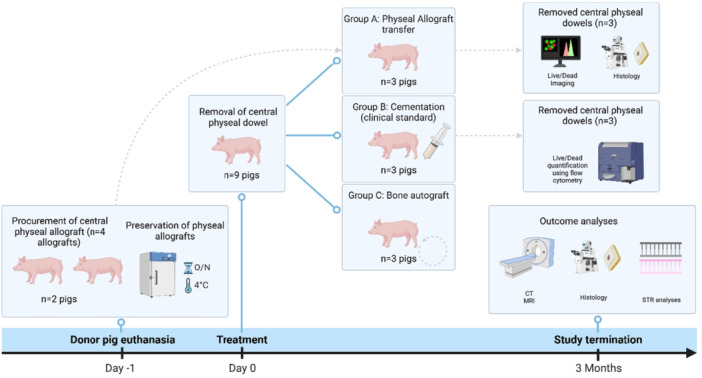
Overview of study groups and procedures. CT, computed tomography; MRI, magnetic resonance imaging; O/N, overnight; STR, short tandem repeat.

This study compares allogeneic physeal transfer (group A) of the distal femur to the current standard treatment (group B, cementation) and filling with autologous bone (group C) in female domestic swine. An overview of the study groups, procedures and outcomes can be found in Figure 1. Upon receiving two donor pigs (males, 39.0 kg and 40.0 kg), 3 recipient pigs (female, 38.0 kg, 40.0 kg, 40.0 kg) were selected based on comparable body weight on the day before the first surgery of surgery to minimize variation between donor and recipient in size and growth rates. Surgeries for the different animals were scheduled on separate days; therefore, while donor–recipient body weights were matched on the day before the first surgery, animals continued to grow between the time of initial selection and surgery. The animals that received cementation (female, 36.0 kg, 40.0 kg, and 41.2 kg) and autologous bone (female, 35.4 kg, 38.2 kg, and 42.2 kg) were allocated randomly by the facility's veterinary support staff. Surgeries were performed per treatment group, starting with group B (cement) on day 1, donors (allograft harvest) on day 2, group A on day 3 (allograft transfer), and group C on day 4 (bone autograft). No blinding was performed in this study, as bone cement is visible on imaging. Although not blinded, the CT scans were performed and analyzed by an investigator from the institutional imaging core facility who was not affiliated with this study or our research group. As this was designed as a safety and feasibility study, the number of animals per treatment group was limited to three animals per group. All animals and data points were used in the final analysis, except for the DNA analysis where one datapoint was missing as no suitable physeal tissue was available of this donor.

### 2.3 Housing and Husbandry

Animals were housed in a dedicated animal care facility accredited by the Association for Assessment and Accreditation of Laboratory Animal Care (AAALAC). Animals were acclimatized for 5–10 days before undergoing surgery. They were housed in groups after surgery and provided with environmental enrichment. Two months after surgery, the animals were moved to a farm facility to ensure sufficient space for the growing pigs. Animal health was monitored daily and when necessary veterinary care was requested.

### 2.4 Surgical Medication and Pain Management

Telazol 5 mg/kg and xylazine 2 mg/kg were administered intramuscularly to induce anesthesia. 1%–3% isoflurane was used throughout the procedure. Buprenorphine 0.03 mg/kg was administered preoperatively as well as rimadyl 4 mg/kg for analgesia. All pigs received a single dose of cefazolin 22 mg/kg intravenous and ceftiofur 5 mg/kg intramuscular during the surgery. For local pain control, 8 mg bupivacaine was infiltrated around the incision site. For post‐operative pain control, rimadyl 4 mg/kg was administered orally daily until lameness resolved. Pigs were euthanized after 3 months follow‐up using pentobarbital intravenously, after telazole 5 mg/kg and xylazine 2 mg/kg intramuscular.

### 2.5 Surgical Procedures

#### 2.5.1 Donor Pigs (*n* = 2)

**Figure 2:**
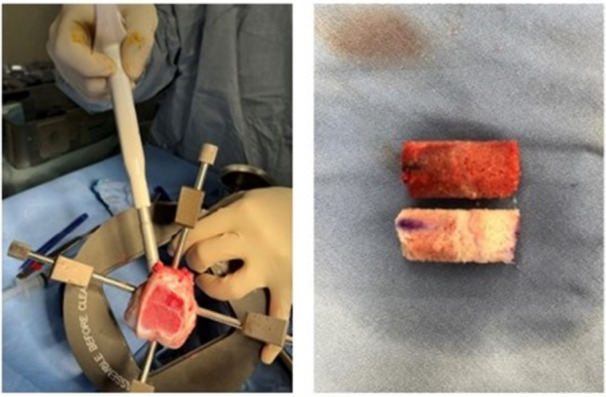
Physeal allograft procurement. Image to the left shows allograft procurement procedure and image to the right shows physeal allograft after overnight preservation (bottom) and removed central physeal dowel from recipient animal. Distal anterior site marked to ensure correct orientation.

After euthanasia, both femurs were dissected. Using an oscillating saw, the trochlear cartilage was dissected to localize the physis. Using an 10 mm Arthrex osteochondral autograft transfer system (OATS) recipient tool, one physis graft was obtained per femur. Allografts were kept overnight in clinical good manufacturing practice (cGMP), chemically defined preservation medium (Dulbecco's Modified Eagle Medium (DMEM), with l‐glutamine, sodium pyruvate, Insulin, Transferrin and Selenium ITS), Non‐essential amino acids (NEAA), l‐Ascorbic Acid, penicillin/streptomycin) at 4°C. Figure 2 shows the physis allograft procurement.

#### 2.5.2 Treated Pigs (*n = *9)

An incision was made on the lateral thigh, through the fascia lata. The vastus lateralis was elevated. A corticotomy of the lateral distal femur was performed and using the 10 mm Arthrex OATS recipient tool, physeal dowel of approximately 25% of the physis was removed. For group A, two pigs underwent surgery on the right distal femur, one on the left. The removed physeal dowel was compared to the graft physis, and the distal end of the allograft was trimmed to match the excised dowel. The allograft was implanted. For group B, two animals underwent surgery on the right distal femur and one on the left. After removal of the physeal dowel, the trajectory was filled with bone cement (Stryker). For group C, two animals underwent surgery on the left femur, and one on the right. After removal of the dowel, it was cut into small fragments using a scalpel and randomly put back in place. For all animals, synovium, muscle and fascia were closed using 0 vicryl, 2‐0 vicryl was used for subcutaneous layers and skin.

### 2.6 Structure and Viability of Physeal Dowels

To assess cell viability after preservation, a pilot study was performed using leftover materials from different animal studies in our center. Allografts were harvested and cell viability was assessed with confocal microscopy (Zeiss) after calcein AM and ethidium homodimer‐1 staining ((Live/Dead® Viability/Cytotoxicity kit; Invitrogen), immediately after harvest or after being placed in preservation medium at 4°C for 24 or 48 h (supplementary data). Viability was quantified using color thresholding on ImageJ.

The physeal dowels removed from group A (Figure 1) were imaged after overnight preservation for a visual confirmation of cell viability. After this, physeal dowels were formalin fixed and embedded in paraffin. Physeal dowels of group B were used for quantification of viable cells using flow cytometry and placed overnight in preservation medium at 4°C. In this case, the next day, dowels were rinsed using pulse lavage for removal of bone marrow components, physes were excised and digested using 0.075% collagenase II (Worthington) in preservation medium at 37°C overnight. Live and dead cells were stained with calcein AM and 7AAD, before quantification with flow cytometry.

### 2.7 Histology

Samples were fixed in neutrally buffered formalin and decalcified using 0.5 M Ethylenediaminetetraacetic Acid (EDTA), samples were dehydrated through graded ethanol steps and embedded in paraffin. Sections were stained with Safranin‐O/Fast Green and immunohistochemistry for type II collagen was performed as previously described. For Tartrate‐Resistant Acid Phosphatase (TRAP) staining, sections were pre‐incubated in 0.2 M acetate buffer with 50 mM tartaric acid at pH 5. After 20 min, 0.5 mg/ml naphtol AS‐MX phosphate and 1.1 mg/ml Fast Red TR salt were added. Sections were incubated for 2 h at 37°C and counterstained with Mayers Haematoxylin.

### 2.8 CT‐Scans

After euthanasia, lower extremities were excised at the proximal femur. Soft tissues were not removed. Complete femurs were scanned on a second‐generation dual‐source CT scanner (SOMATOM Definition Flash, Siemens Healthineers GmbH, Forchheim, Germany). The scans were performed using a routine extremity protocol with a detector collimation of 64 × 0.6 mm, tube potential of 120 kV, and a dose of 46 mGy CTDIvol. The corresponding CT images were reconstructed using an iterative reconstruction algorithm (SAFIRE, strength = 3) with Br60 and Qr51 convolution kernels, a 512 × 512 pixel matrix, and a slice thickness and increment of 0.5 mm. Additionally, the distal femur was scanned using an ultra‐high resolution extremity protocol with detector collimation of 16 × 0.3 mm, tube potential of 120 kV, and a dose of 93 mGy CTDIvol. The corresponding CT images were reconstructed using the same iterative reconstruction algorithm (SAFIRE, strength = 3) with Ur70 and Ur77 convolution kernels, a 512 × 512 pixel matrix, and a slice thickness and increment of 0.4 mm.

Three‐dimensional (3D) reconstructions of both femora were made from femur CTs. The right femur was mirrored. The operated femur was projected onto the unoperated contralateral side femur using a best fit on the proximal end of the femur to visualize potential deformation after treatment. Femur length was measured from the most proximal to most distal location.

Mineralized bone was segmented and volume of mineralized bone in the complete femur was measured for each femur. Total bone volume was defined as the volume enclosed by the periosteal surface of the cortical bone and was measured for each femur. A calibration phantom was scanned to establish the relationship between Hounsfield units (HU) and bone mineral density (BMD, g/cm³), enabling post‐hoc conversion of the HU values to BMD values.

### 2.9 MRI‐Scans

T1 weighted images (T1), T2 weighted fat‐suppressed images (T2 FS), and spoiled gradient echo sequences (SPGR) were obtained using a 3.0 T MRI scanner (GE healthcare, Chicago, Illinois, USA) with a slice thickness of 2 mm. For T2 FS, a TR (repetition time) of 3322–3346 ms was used and a TE (echo time) of 39–50 ms with a flip angle of 111°. For T1, TR of 737–742 ms was used and TE of 9.13–9.36 ms and a flip angle of 111°. SPGR sequences were obtained with TE of 200 ms and TE of 4.86 ms and a flip angle of 70°.

### 2.10 DNA Analysis

Tissue samples were obtained from two out of the three transferred physes after euthanasia. Ear biopsy of donor and recipient pigs were used as reference material. Short Tandem Repeat analysis (STR) was performed by Van Haeringen Laboratorium (Wageningen, the Netherlands), to determine presence of donor DNA in the transferred physis. This technique detects donor DNA contributions exceeding 5% of the total DNA isolated from the grafted region.

### 2.11 Data Analysis and Statistics

Given the small sample size in this safety and feasibility large‐animal study, we prioritize transparent reporting of individual outcomes. For quantitative data, we present all individual animal data points (per subject) in the figures, tables, and text. Descriptive statistics (mean and SD/SEM where indicated) are provided for context only and are not used to support claims. No statistics were performed due to limited sample size and the exploratory aims of this work. Where applicable, figure legends specify the exact n, the unit of analysis, and how each data point was derived. Viability is shown as average with standard deviation.

## 3 Results

Creation of a central physeal defect and subsequent surgical treatment

No complications occurred during surgery. All animals recovered well from surgery without severe clinical lameness. One pig of group A and one of group B underwent surgical drainage of an abscess, and were treated antibiotically for surgical site infection. One animal of group A was treated for pityriasis rosea. All animals survived until the final follow‐up without graft removal or severe lameness.

### 3.1 Transfer of Intact and Viable Physis

Surgical feasibility was confirmed using leftover tissue from unrelated studies in the same species in our center, using the same experimental procedures. These pilot experiments assessed cell viability immediately after procurement and after 24–48 h of preservation (see Supporting Information: Figure S1) using quantification of live/dead imaging.

**Figure 3:**
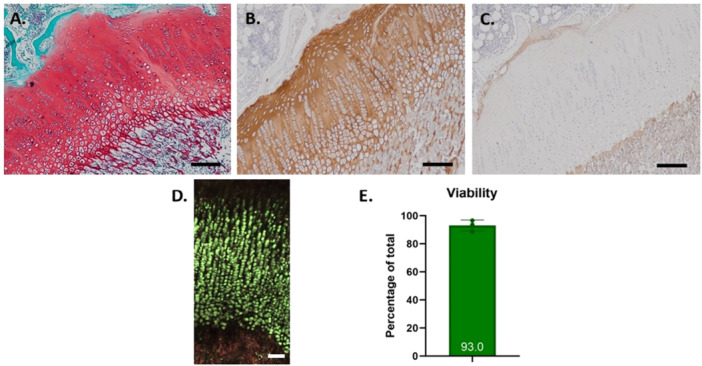
Morphology and viability of physis at time of implantation. (A) Safranin‐O/Fast Green staining demonstrating glycosaminoglycan‐rich cartilage matrix in the physis. (B) Type II collagen immunohistochemistry, showing the typical cartilaginous extracellular matrix. (C) Type VI collagen immunohistochemistry, demonstrating most pronounced type VI collagen pericellular in the resting zone and at the transition of cartilage to bone. (D) Live/Dead staining using ethidium homodimer‐1 (non‐viable cells, red) and calcein AM (viable cells, green), imaged with confocal microscopy. Viable cells are clearly demarcated in the resting, proliferative, and hypertrophic zones (green), whereas the mineralized zone shows diffuse red background signal without distinct nuclear morphology, consistent with nonspecific EthD‐1 binding to DNA remnants in calcified matrix. (E) Flow cytometry quantification of cell viability after enzymatic digestion of 3 allografts from different donors, stained using calcein AM (live) and 7‐aminoactinomycin (7‐AAD). Data are expressed as the percentage of living cells relative to total cells. Scale bar = 200 µm.

In the current study, physeal dowels from the pigs that underwent subsequent treatment were removed using the same technique as the allografts, and could thus be used to confirm viability and histology of the allografts. Using this surgical approach we were able to excise an intact physis (Figure 3). Viability of the physeal tissue was 93.0 ± 3.9% after overnight preservation and subsequent tissue digestion, as measured by flow cytometry.

### 3.2 Femur and Physis Morphology

**Figure 4:**
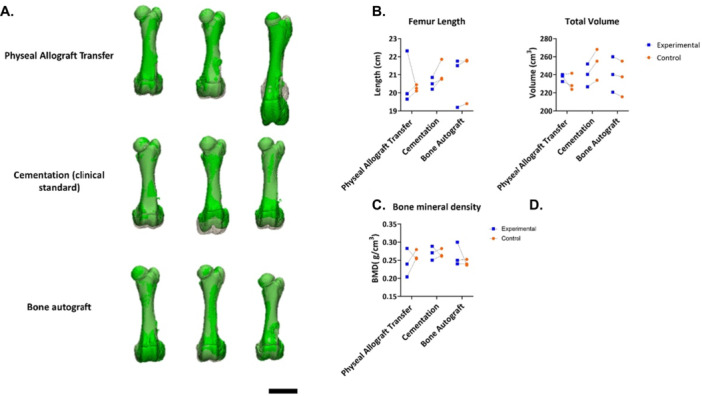
Computed tomography (CT) evaluation of experimental and contralateral femurs at 3‐month follow‐up. (A) Three‐dimensional reconstructions of treated and contralateral femurs. The treated femur (green) is projected onto the contralateral control using a proximal best‐fit alignment after mirroring one femur to allow direct comparison. Scale bar = 5 cm. (B) Femur length as measured from CT reconstructions between the most proximal and distal points of the bone. (C) Total outer bone volume, defined as the volume enclosed by the cortical bone surface mask. (D). Bone Mineral Density (gram/cm^3^) Individual data points per donor are shown in the graphs.

**Figure 5:**
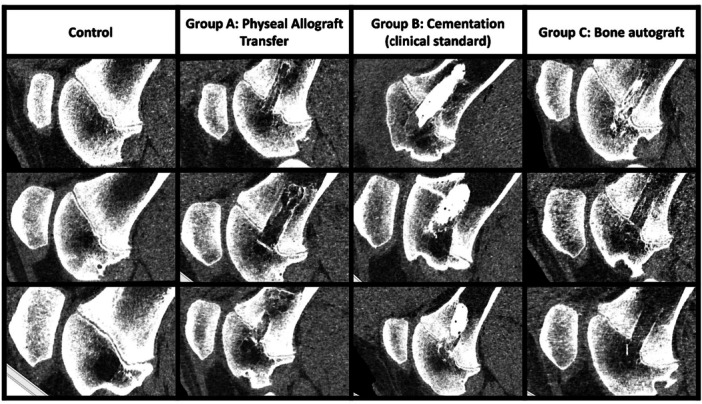
Ultra high‐resolution computed tomography after 3 months follow‐up. Representative CT reconstructions of the distal femur after 3‐month follow‐up. In the untreated control limb, a thin radiolucent physis and radiopaque metaphysis are visible, with central cystic changes in the epiphyseal area. In Group A (physeal allograft), a central hypodense region bridging the physeal zone indicates low bone density at the graft site, without evidence of bridging callus. In Group B (cementation), radiopaque cement material is retained within the physeal defect or proximal the epiphyseal area (lower image). In Group C (autologous bone graft), one animal demonstrated axial trabecular bone formation within the defect (upper image), while the other two showed limited bone density consistent with resorption. Images shown for all experimental animals and one representative control.

**Figure 6:**
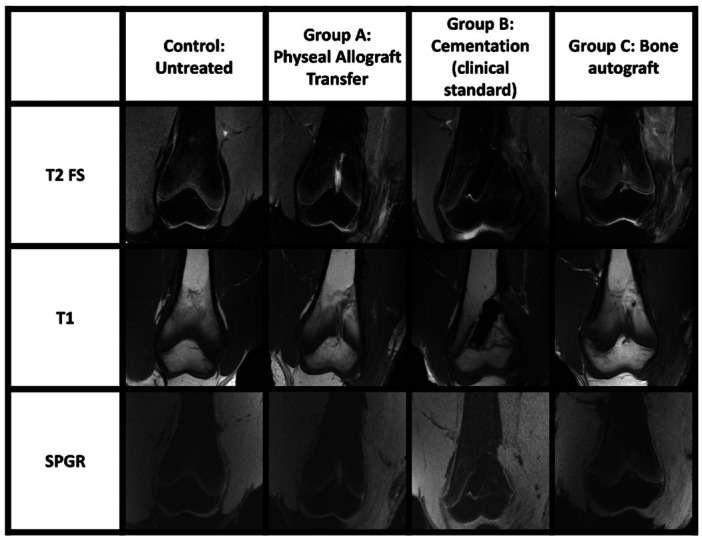
Magnetic Resonance Imaging of treatment groups and untreated control after 3 months follow‐up. Representative T2‐weighted Fat‐Suppressed (T2FS), T1‐weighted (T1), and Spoiled Gradient Recalled (SPGR) sequences. T2FS images (TR 3322–3346 ms, TE 39–50 ms, flip angle 111°) revealed edema and fluid accumulation surrounding the transferred allograft and adjacent to the bone cement. T1‐weighted images (TR 737–742 ms, TE 9.1–9.4 ms, flip angle 111°) demonstrated graft areas with signal intensity similar to fat in the bone autograft group, with no evidence of physeal bar formation. SPGR images (TR 200 ms, TE 4.9 ms, flip angle 70°) did not distinguish between fluid and physeal cartilage. These images are representative for 3 animals per group (3 operated femurs, 3 control contralateral femurs).

In two of the three animals that underwent allogeneic physeal transfer, the operated femur was 0.5 cm shorter compared to the contralateral limb length, whereas in the third animal the operated femur was 2.0 cm longer than its contralateral counterpart (Figure 4A and B). In group B, the femurs that received bone cement were 0.3 cm shorter, and 1.0 cm shorter, and 0.6 cm shorter than the contralateral femur. In the group that received autologous bone grafts (group C), operated and control femurs were of the same length, 0.3 cm shorter, and 0.2 cm shorter. Figures 4C and 4D demonstrate the mineralized bone volume (also including the placed bone cement) and total volume of all experimental and control femurs. High resolution CT scans (Figure 5) demonstrate low bone density around the operated site of transferred allografts, indicating bone resorption. The radiopaque bone cement is in place. No bony bars are visible in any of the CT scans. MRI (Figure 6) show edema/fluid around the transferred allograft and around the bone cement. The bone autograft shows signal intensity comparable to fat in the graft area, and no signs of bone bar formation. There is no continuity of the physis in any of the groups. In addition, cystic changes were identified in both the treated and the untreated limbs (Figure 5 and Figure S1).

### 3.3 Histologic Appearance

**Figure 7:**
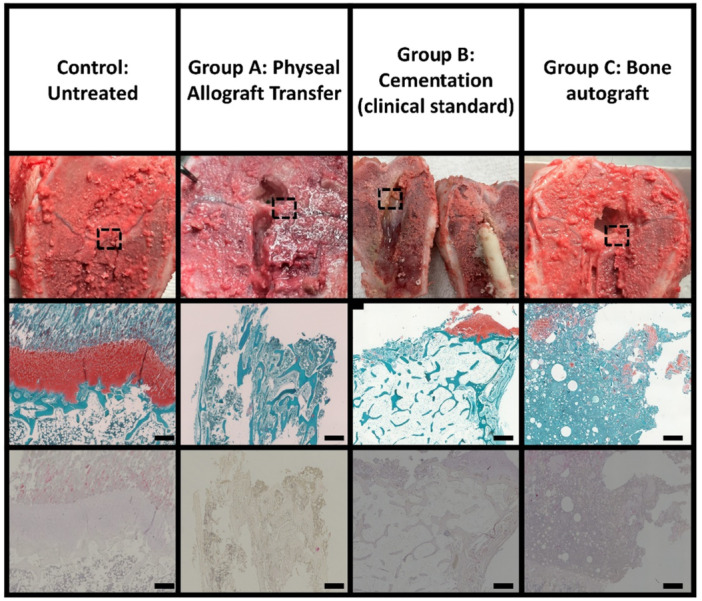
Histological appearance of treated physeal defects compared with untreated controls. For each animal, the contralateral limb served as the untreated control, and two sections of the operated region were analyzed. Representative control sections demonstrate organized growth plate architecture with strong safranin‐O staining for glycosaminoglycans in the physis and tartrate‐resistant acid phosphatase (TRAP) staining (red/pink) along trabecular surfaces. In contrast, treatment groups display disorganized tissue morphology, with sporadic/localized regions resembling physeal organization and extensive cyst formation in treated samples, the images shown are representative of the observed findings. Scale bar = 200 µm.

The untreated control shows well‐organized tissue architecture in the physis with positive Safranin‐O staining indicating glycosaminoglycans (Figure 7). Additionally, positive Tartrate‐Resistant Acid Phosphatase (TRAP) staining is observed around the trabecular surfaces, appearing red. This TRAP staining indicates active osteoclastic activity involved in the endochondral ossification. In contrast, the treatment groups display disorganized tissue architecture, suggesting an altered or incomplete repair response within the physeal defect. While small regions show some resemblance to native physeal tissue, evident in scattered areas that mimic the glycosaminoglycan‐rich extracellular matrix, the lack of organized structure across the defect site suggests impaired or disrupted physeal regeneration.

### 3.4 No Donor DNA Detectable in Transferred Physis

Three months after physeal allograft transfer, DNA present in the two physes that were tested contained only recipient DNA and no donor DNA. Insufficient DNA was available of physeal tissue of the third recipient.

## 4 Discussion

This study demonstrates the feasibility of a physeal allograft transfer in a large animal model. We demonstrate successful procurement of physeal allograft dowels and sustained high viability after overnight preservation. Furthermore, no severe adverse reactions and no gross deformation of the operated femurs were found within 3 months after surgery. However, the physeal allografts did not show integration into host tissue, multiple cysts were observed after 3 months, and no donor DNA was detected at 3 months, indicating no persisting functional physeal cells are present after 3 months. Unexpectedly, no physeal bar was formed in the autograft group, which limits current study's capacity to distinguish between treatment effects and natural variability.

The lack of physeal bar formation may reflect individual variability, as this study is limited by a small sample size (n = 3 per group), or the follow‐up period might have been too short to enable bar formation. However, histologic signs of bridging were evident by 3 weeks following fracture in experimental studies in rats [12], suggesting that 3 months follow‐up is sufficient for bar formation. Additionally, we created a central physeal defect of approximately 25% of the size of the physis. Defects exceeding 20% of the physis result in gross deformity in sheep [13], and defects located in the center of the physis often result in limb length discrepancy. Current study allowed for 3 months follow‐up, during which approximately 4 cm axial growth is expected in the femur [11]. Notably, we did not observe any growth deficiency exceeding 1 cm in any of the treatment or control groups, indicating that the growth from surrounding persistent physis is sufficient to prevent severe growth deficiency. Similarly, others have shown that growth plate ablation results in on average 1 cm length difference between femurs of pigs in 2‐4 months follow‐up [14].

The implanted physeal allograft did not integrate into surrounding physis and did not show structural physeal organization. This lack of integration or physeal organization could indicate graft rejection, or a result of ischemic changes in the growth plate as we did not vascularize the graft. In addition, as we used a ‘press‐fit’ to fix the graft into the defect size, which might have caused damage by compression or crushing of the physis. Follow‐up studies should evaluate using different graft placement techniques. In contrast to our findings, ischemic injury to the growth plate resulted in a total absence of growth and the replacement with bone and fibro‐fatty tissue of the physeal area in a rabbit model [15]. The bone resorption in the cysts that were observed could have prevented the formation of a bony bar. The formation of cysts might be the result of immune reactions to the allogeneic type II collagen [13, 16], but are more likely inherent to the pig animal model as were also found in the contralateral non‐operated femurs, and in the femurs treated with autologous bone grafts. Clinically, the cystic changes did not result in adverse reactions or lameness, but the long‐term functional consequences of such cystic alterations remain uncertain. In the current study, we located the defect purposely outside of the joint to reduce joint‐morbidity and facilitate surgical access, but this anatomical location might have shielded the grafts from mechanical loading. The lack of mechanical loading could impair osteogenesis and lead to decreased bone density and structural integrity. Future studies could consider a different surgical approach.

Interestingly, one of the femurs that received a growth plate allograft was 2 cm longer than the contralateral femur. This is in line with findings of vascularized physeal allograft transfer in rabbits, where the increased growth of transferred femurs was attributed to increased blood flow due to surgery [17]. In addition, it could be the result of excessive bone formation as caused by inflammation [18], as this concerned the animal that suffered from postoperative wound infection. Lastly, we demonstrated high viability of explanted pig allografts over short preservation periods, similar to rabbit allografts [19]. Surprisingly, no donor DNA was found at the location of the transferred allografts, indicating that the cells from the donor graft did not survive 3 months after transfer. These findings may result from immune‐mediated clearance, or ischemic cell death.

As this study represents an early‐stage investigation into the safety and feasibility of physeal allograft transfer (PAT) in a large animal model, several limitations must be acknowledged. The study was designed with a small sample size as it represents the first investigation of this intervention in a large animal model. The primary aim was to determine feasibility and gather foundational data to inform future adequately powered studies. Given the novelty of the approach and the invasive nature of the procedures, it is not ethically justifiable to use a larger number of animals without demonstration of feasibility. Our control groups were designed to reflect clinically relevant comparators. Most notably, this study is limited by the failure of the autologous bone group to develop physeal bars. This compromises the intended role of this group as a functional positive control. Although this study was not designed or powered to allow statistical comparison between groups, this limitation does underscore the need for establishing a reliable control group before large‐scale studies are pursued. Furthermore, no blinding and incomplete randomization were performed and data should be interpreted in light of these design constraints.

This study did not include serial growth measurements such as radiographs. Repetitive radiographs would require anesthesia and cause severe animal discomfort. Instead, this study relied on terminal measurements and used the contralateral, non‐operated limb of each animal as an internal control for growth. This approach assumes normal growth in the contralateral limb, and it is theoretically possible that both limbs experienced reduced growth during the study period. However, based on the animals’ age and published growth data (4 cm) [11], continued growth would be expected over the 3‐month follow‐up. Future studies could incorporate serial quantitative imaging to track growth dynamics directly, or include deformation indices. Moreover, we prioritized endpoint histology in this study and recommend that more invasive longitudinal viability assessments be considered only after establishing a robust and reproducible model. As no quantitative and validated histological score is available in the literature, our analysis was qualitative. Development and validation of histological scoring systems of physeal allograft integration is recommended for future studies.

No immunological assays were measured to evaluate host immune response to the allograft. While systemic inflammatory blood markers are elevated after surgery in general, the inclusion of control groups (cement, autograft) and serial blood sampling during the study could have facilitated distinction between generic post‐surgical inflammation and graft‐specific immune responses, although statistical differentiation might have been limited due to the small sample size. Additionally, attempts to perform immunohistochemical staining for immune cell infiltration (CD3, CD8, and CD68) were unsuccessful, likely due to the extended decalcification process required for bone tissue processing, and the loss of native tissue architecture compromising the interpretation of data. Future studies should incorporate comprehensive immunological assessment including serial blood sampling for inflammatory markers and cytokine profiling, optimized tissue processing protocols to preserve antigens for immune cell staining, and sample size to better characterize host immune responses to the allograft.

Lastly, we transplanted female pigs with male allografts and matched based on weight. Growth rate differences and sex‐differences might have influenced our outcomes, and the influence of these factors should be studied in follow‐up experiments.

Despite these limitations, this study provides valuable preliminary insights. We successfully established a surgical workflow, confirmed the viability of pediatric donor physes post‐preservation, and studied femur morphology over time. As such, this study serves as a critical pilot investigation to inform subsequent research, rather than a definitive efficacy trial.

## 5 Conclusions

This is the first report of viable physeal allograft preservation and subsequent transfer in a large animal model. High viability of the allografts and structural integrity after procurement underline the potential of this treatment. However, transferred physes demonstrate poor integration and incomplete repair response. The domestic pig animal model seems inadequate to assess clinical success of allograft transfer due to the lack of bone bar formation.

## Author Contributions

Study Design: J. V. Korpershoek, T. A. Milbrandt, M. L. Floren, D. B. F. Saris, A. N. Larson; Data acquisition: J. V. Korpershoek, C. Chen, C. V. Nagelli, K. L. Lydon; Analysis and interpretation of data: J. V. Korpershoek, T. A. Milbrandt, M. L. Floren, D. B. F. Saris, A. N. Larson; Drafting of the manuscript: J. V. Korpershoek; Reviewing and final approval of the manuscript: C. Chen, C.V. Nagelli, K. L. Lydon, T. A. Milbrandnt, M. L. Floren, D.B.F. Saris, A. N. Larson, T. A. Milbrandt.

## Supporting information

Supplemental.docx.

## Data Availability

The MRI and CT data supporting the findings of this study will be made available upon reasonable request to the corresponding author.
