# Supplementary material for: Physeal Allograft Transfer for Physeal Bars: A Safety and Feasibility Study in a Domestic Swine Model
Source: J Orthop Res. 2026 Jan 4;44(1):e70133. doi: 10.1002/jor.70133 (PMC12765232; doi:10.1002/jor.70133)
Supplement: Supplementary file 1 — Supplemental.docx. [file JOR-44-na-s001.docx]

# Supplemental information:

| **Treatment group** | **Weight at surgery** (in kg) |
| --- | --- |
| Donor | 39 |
| Donor | 40 |
| Transplantation | 40 |
| Transplantation | 38 |
| Transplantation | 40 |
| Cement | 41.2 |
| Cement | 36 |
| Cement | 40 |
| Autologous bone | 42.2 |
| Autologous bone | 38.2 |
| Autologous bone | 35.4 |

**Table S1:** Treatment groups and weight at time of surgery.

**Institutional humane endpoints**:

- Weight loss greater than or equal to 20% of body weight
- Inability to ambulate
- Inability to reach food and/or water
- Tumors greater than or equal to 10% body weight
- Tumors that have ulcerated
- A body condition score of 1 or less using the IACUC approved scoring system

**Additional humane endpoints:**

- Osteomyelitis or septic arthritis unresponsive to treatment
- Grade 3-4 lameness unresponsive to analgesic treatment


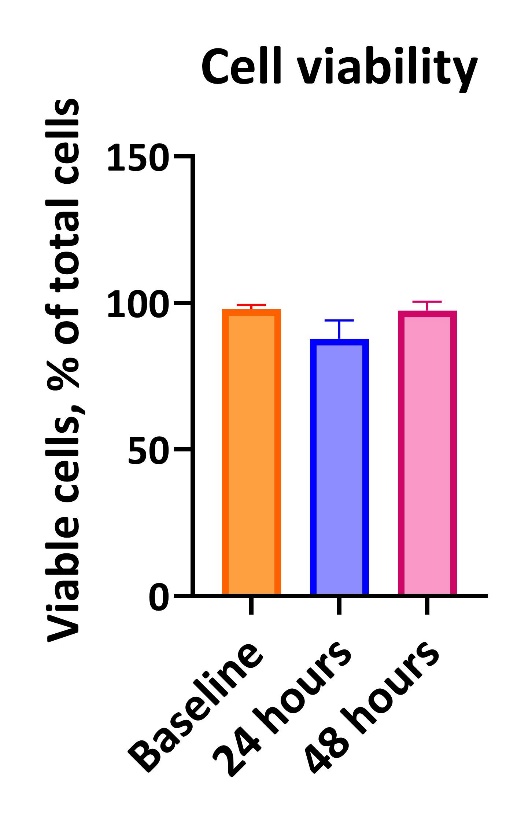


**Figure S1. Viability of physeal allografts immediately after procurement and following preservation.** Physeal allografts (10-mm diameter) were harvested using an osteochondral autograft transfer system. Viability was assessed at baseline (immediately post-procurement) and after 24 and 48 hours of storage in preservation medium. Samples were stained with **calcein AM (viable cells, green)** and **ethidium homodimer-1 (non-viable cells, red)** using a Live/Dead® kit (Invitrogen) and imaged with a Zeiss confocal microscope. In total, 2-4 images were taken per allograft of two allografts from 2 different donors. Quantification of viable cells was performed in ImageJ by applying color thresholding to calculate the proportion of calcein-positive cells relative to total signal.


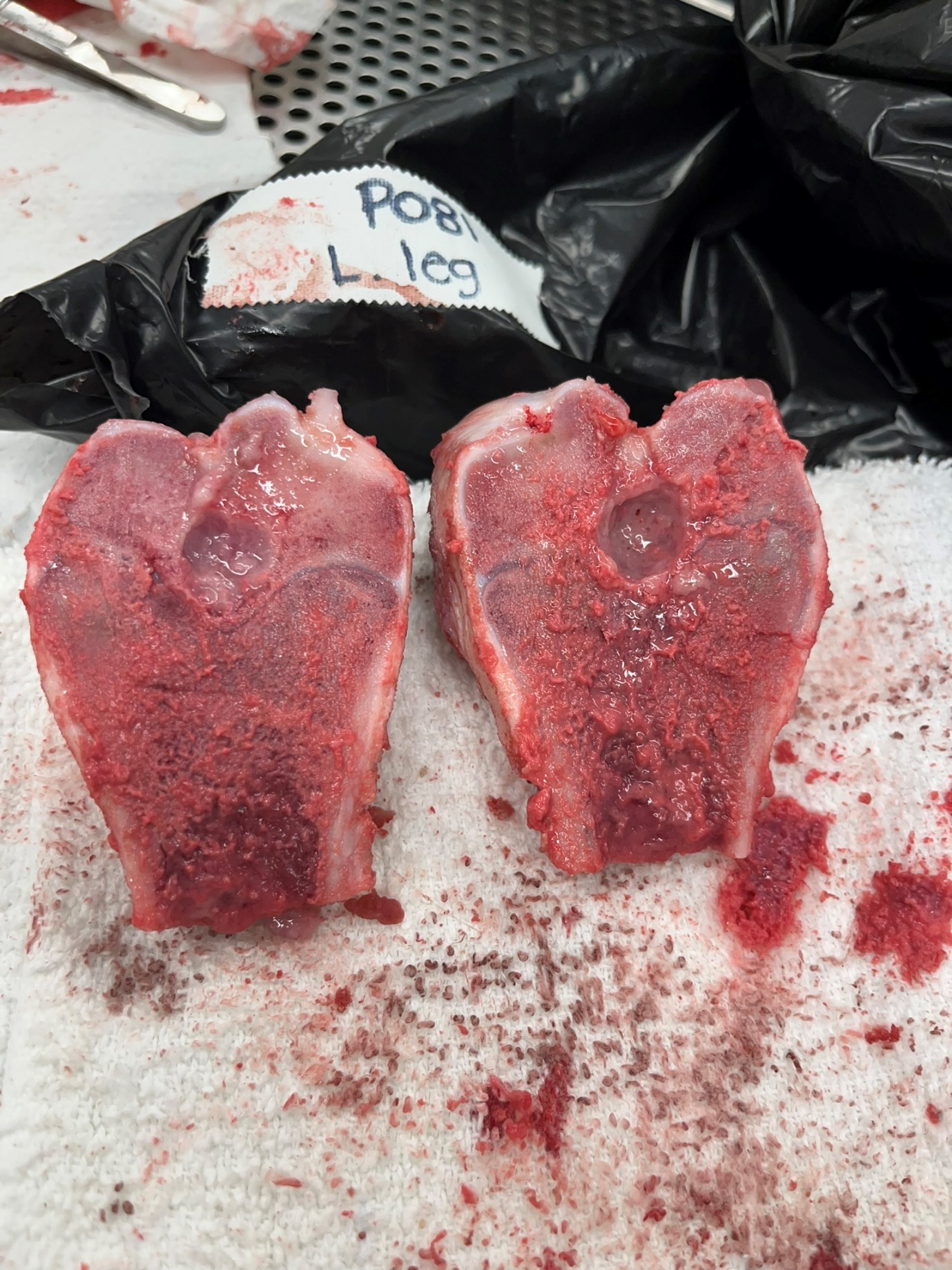


**Figure S2.** Cystic changes in nontreated distal femur.
